# Diagnosis is not enough: Selective behavior change after chronic disease onset in Thailand

**DOI:** 10.1371/journal.pone.0360648

**Published:** 2026-10-30

**Authors:** Ruttiya Bhula-or, Attakrit Leckcivilize

**Affiliations:** 1 Labour Research and Coordination Research Unit, College of Population Studies, Chulalongkorn University, Bangkok, Thailand; 2 Nuffield Department of Medicine, University of Oxford, Oxford, United Kingdom; All India Institute of Medical Sciences, INDIA

## Abstract

Health shocks—such as new diagnoses of chronic disease—can disrupt daily life and create potential “teachable moments” for behavioral improvement. Yet, existing evidence on post-diagnosis behavior change remains mixed and largely limited to high-income countries. This study addresses a major empirical gap by examining whether chronic disease diagnoses are associated with measurable within-person changes in health behaviors among middle-aged and older adults in Thailand, a rapidly aging middle-income society. We analyzed four waves (2015–2022) of the *Health, Aging, and Retirement in Thailand* (HART) survey. The analytic sample comprised adults aged ≥45 years with no baseline diagnosis of cancer, diabetes, or cardiovascular disease (N = 2,891; 9,577 person-wave observations). Self-reported physician-diagnosed conditions were used to construct disease-status indicators. Behavioral outcomes included smoking, current alcohol use, sufficient physical activity (≥150 minutes/week), normal body mass index (BMI) (18.5–23), and waist obesity (sex-specific cutoffs). Individual fixed-effects linear probability models with survey-wave fixed effects and adjustment for time-varying covariates (log income, activities of daily living [ADL] score, and marital status) estimated within-person associations, with standard errors clustered at the individual level. Disease-specific models and models including interactions with sex assessed heterogeneity. In pooled analyses, the disease-status indicator was not significantly associated with smoking, alcohol use, physical activity, BMI, or waist obesity. In contrast, a diabetes diagnosis was associated with a reduction of 3.6 percentage-point in current alcohol use, and both diabetes and cancer diagnoses were associated with reductions in frequent drinking. Models including interactions with sex suggested larger diagnosis-associated reductions in selected alcohol-use outcomes among men, although statistical evidence varied by outcome and disease type. Among middle-aged and older Thai adults, chronic disease diagnoses were associated with selective, rather than comprehensive, changes in health behaviors. Diagnosis alone is insufficient to support changes across multiple health behaviors, underscoring the need for structured post-diagnosis counselling, follow-up, and gender-sensitive secondary prevention in aging middle-income populations.

## Introduction

Non-communicable diseases (NCDs) account for a substantial share of the global burden of mortality and disability, with modifiable risk factors including tobacco use, alcohol consumption, poor diet, and physical inactivity [1]. A new diagnosis of cancer, diabetes, or cardiovascular disease may act as a health shock that prompts individuals to change these behaviors. However, longitudinal evidence on post-diagnosis behavior change remains mixed and derives largely from high-income countries [2].

In Asia, studies from South Korea report persistent smoking and physical inactivity among people with chronic disease [3,4], alongside limited dietary improvements after diagnosis [5]. Chinese longitudinal evidence links health shocks to reductions in smoking and alcohol use, but findings for exercise and preventive care are inconsistent [6]. Evidence from Southeast Asia remains sparse; research in Cambodia identifies structural and psychological barriers to sustained change [7]. These findings underscore the need for further longitudinal analysis in middle-income Southeast Asian settings.

Thailand provides a relevant setting because of its rapid population aging and growing burden of chronic disease. Despite universal health coverage and health-promotion initiatives, behavioral risks remain a concern; research among Bangkok residents, for example, documents patterns of physical activity and sedentary behavior [8]. Understanding whether diagnosis is associated with changes in health behaviors can inform post-diagnosis support for middle-aged and older adults.

The Health, Aging, and Retirement in Thailand (HART) survey provides repeated observations of health and behavior among the same individuals over time, allowing within-person comparisons across waves. This design addresses a key limitation of cross-sectional research, in which observed associations between diagnosis and behavior may simply reflect stable differences between individuals rather than behavioral changes following diagnosis.

Health behavior theories identify risk perception, motivation, and readiness to change as potential mechanisms linking diagnosis to behavioral change [9], while integrative socio-ecological frameworks emphasize that social relationships, health-system engagement, and environmental constraints also shape whether a “teachable moment” translates into action [10]. These perspectives guide our interpretation of whether, and for whom, behavioral responses to diagnosis differ across outcomes.

Using four HART waves (2015, 2017, 2020, and 2022), this study examined within-person changes in health behaviors associated with diagnoses of cancer, diabetes, and cardiovascular disease, including differences by disease type and gender. Two-way fixed-effects models estimated contemporaneous associations across waves in which diagnoses were reported, including new and ongoing diagnoses.

### Theoretical background and literature review

Health behavior theories provide useful frameworks for understanding how individuals perceive health risks, respond to chronic disease diagnoses, and sustain lifestyle changes. The Health Belief Model (HBM) emphasizes perceived susceptibility, severity, benefits and barriers, alongside cues to action [11]. Within this framework, diagnosis may be interpreted as a cue to action, potentially increasing perceived vulnerability and motivation [12]. The Transtheoretical Model (TTM) conceptualizes behavior change as a staged and cyclical process, involving stages such as precontemplation to contemplation, preparation, action, and maintenance [13]. Because individuals differ in their readiness to change, interventions can be tailored to their stage of change [9]. Research on older adults’ physical activity further highlights the importance of social support, community connections and environmental conditions, indicating the value of examining individual motivation within its social context [14].

Scholars increasingly advocate multi-theoretical and multilevel approaches that integrate motivational, relational, and environmental dimensions. Self-Determination Theory and Social Cognitive Theory, for example, have been combined in an integrated model linking autonomous motivation, behavioral intentions, and perceived control [15]. Similarly, Strong Structuration Theory links individual practices to broader institutional and structural conditions, helping explain why diagnosis alone may not be sufficient to produce lasting behavioral change [16].

A chronic disease diagnosis is often conceptualized as a health shock or “teachable moment” that can prompt lifestyle adjustment. Yet empirical evidence remains mixed. A longitudinal study in the United States reported reduced substance use and increased utilization of preventive medical procedures following chronic disease diagnoses, although physical activity declined after diagnoses of lung disease, cancer, and stroke [12]. Evidence from Korea shows that men with type 2 diabetes were more likely to continue smoking if they were younger, had lower economic status, or heavier smokers before diagnosis [3]. In China, a cross-sectional study found that tobacco-related chronic diseases were associated with smoking cessation among smokers in a rural area of Shanghai [17].

Research on smoking cessation highlights the potential relevance of psychosocial and behavioral support. Cognitive behavioral approaches aim to modify dysfunctional thought and behavior patterns [18], while a prospective cohort receiving treatment incorporating nurse counselling identified self-efficacy and desire to smoke as factors associated with initial quitting and subsequent maintenance over 12 months [19]. Evidence from Indonesia also shows that cessation-stage classifications among school-going adolescents are associated with family and environmental factors, including parental smoking, school smoking exposure, and tobacco marketing [20].

The literature suggests that while a chronic disease diagnosis can initiate meaningful health behavior change, the sustainability of these changes may be influenced by psychosocial support and social and environmental conditions [10,14,19].

## Methods

### Data source

This study aimed to estimate within-person changes in modifiable health behaviors associated with reported chronic disease diagnoses (“health shocks”) among middle-aged and older adults in Thailand. We conducted a longitudinal observational panel analysis, using four waves of data from the HART longitudinal survey in 2015, 2017,2020, and 2022 [21–24]. The survey used a multi-stage, stratified probability sampling approach covering six regions, with provinces outside Bangkok and its vicinity selected using probability proportional to size (PPS), where size referred to the provincial population. All four provinces in Bangkok and its vicinity were included with certainty. District selection was followed by random sampling of eligible individuals from population registers. The dataset was accessed for research purposes on [15 August 2025]. The authors accessed de-identified data and did not have access to information that could identify individual participants during or after data access. The HART survey collected repeated information on health status, chronic disease diagnoses, health behaviors, healthcare utilization, and socioeconomic characteristics of Thai adults aged 45 and older. Data were drawn from Waves 1–4 (2015, 2017, 2020, and 2022). To assess behavior change in response to new health shocks, we included individuals who participated in at least two consecutive waves and reported no prior diagnosis of cancer, diabetes, or cardiovascular disease at baseline. This restriction allowed us to identify within-person behavioral responses to new, incident diagnoses. The descriptive sample comprised 2,891 individuals and 9,577 person-wave observations (Table 1). Regression samples varied by outcome, ranging from 9,164–9,537 person-wave observations (Table 2).

**Table 1:** Descriptive statistics.

	wave				
	2015	2017	2020	2022	Total
N	2,595 (27.1%)	2,891 (30.2%)	2,234 (23.3%)	1,857 (19.4%)	9,577 (100.0%)
Any 3 diseases	0.000 (0.000)	0.090 (0.286)	0.142 (0.349)	0.165 (0.371)	0.092 (0.289)
Cancer	0.000 (0.000)	0.010 (0.098)	0.015 (0.121)	0.016 (0.126)	0.010 (0.097)
Diabetes	0.000 (0.000)	0.052 (0.221)	0.091 (0.287)	0.095 (0.293)	0.055 (0.228)
Cardiovascular	0.000 (0.000)	0.031 (0.174)	0.043 (0.204)	0.064 (0.244)	0.032 (0.176)
Female	0.515 (0.500)	0.523 (0.500)	0.547 (0.498)	0.551 (0.497)	0.532 (0.499)
Age	65.58 (11.95)	67.91 (12.05)	69.63 (11.42)	71.79 (10.96)	68.43 (11.88)
Log personal income	9.994 (2.987)	4.597 (5.090)	9.133 (3.349)	9.383 (2.625)	8.046 (4.408)
ADLscore (four items)	4.087 (1.005)	4.289 (1.554)	4.328 (1.631)	4.532 (2.425)	4.290 (1.669)
Separated	0.019 (0.136)	0.013 (0.115)	0.018 (0.134)	0.016 (0.124)	0.016 (0.127)
Divorce	0.025 (0.156)	0.031 (0.173)	0.029 (0.167)	0.032 (0.176)	0.029 (0.168)
Widow	0.284 (0.451)	0.270 (0.444)	0.301 (0.459)	0.324 (0.468)	0.291 (0.454)
Single	0.054 (0.225)	0.060 (0.238)	0.053 (0.225)	0.052 (0.222)	0.055 (0.228)
No education	0.064 (0.244)	0.061 (0.239)	0.051 (0.219)	0.047 (0.212)	0.057 (0.231)
Secondary education	0.127 (0.333)	0.159 (0.365)	0.138 (0.345)	0.137 (0.344)	0.141 (0.348)
Diploma or higher	0.050 (0.217)	0.046 (0.209)	0.039 (0.193)	0.033 (0.180)	0.043 (0.202)
Rural Area	0.496 (0.500)	0.507 (0.500)	0.528 (0.499)	0.504 (0.500)	0.508 (0.500)
Smoking	0.140 (0.347)	0.151 (0.358)	0.129 (0.335)	0.118 (0.323)	0.136 (0.343)
Alcohol	0.140 (0.347)	0.194 (0.395)	0.141 (0.348)	0.141 (0.348)	0.157 (0.364)
Enough Exercise	0.217 (0.413)	0.298 (0.458)	0.253 (0.435)	0.227 (0.419)	0.252 (0.434)
Normal BMI	0.393 (0.488)	0.402 (0.490)	0.386 (0.487)	0.400 (0.490)	0.395 (0.489)
Waist obese	0.415 (0.493)	0.414 (0.493)	0.298 (0.458)	0.363 (0.481)	0.377 (0.485)

Note: Continuous variables are presented as mean (standard deviation), and binary variables as proportion (standard deviation). The N row reports person-wave counts and their percentages of the pooled total.

**Table 2:** Association between chronic disease diagnosis and health behaviors.

			Enough	Normal	Waist
VARIABLES	Smoking	Drinking	Exercise	BMI	Circumference
Any 3 diseases	−0.009	−0.023	0.017	0.026	−0.025
	(0.0128)	(0.0144)	(0.0210)	(0.0208)	(0.0203)
log personal income	0.002**	0.002**	0.001	−0.001	−0.006***
	(0.0007)	(0.0010)	(0.0014)	(0.0013)	(0.0013)
ADL score (four items)	−0.005**	−0.004***	−0.017***	−0.000	−0.004
	(0.0024)	(0.0016)	(0.0024)	(0.0041)	(0.0036)
separated	−0.027	−0.044	−0.015	−0.031	0.055
	(0.0250)	(0.0325)	(0.0528)	(0.0487)	(0.0423)
divorce	−0.046*	−0.023	0.012	0.041	0.029
	(0.0255)	(0.0320)	(0.0444)	(0.0448)	(0.0409)
widow	−0.017	−0.015	−0.014	0.043	−0.028
	(0.0139)	(0.0151)	(0.0248)	(0.0275)	(0.0239)
single	−0.015	−0.041	−0.029	0.049	−0.077*
	(0.0324)	(0.0383)	(0.0538)	(0.0600)	(0.0468)
wave 2	0.026***	0.073***	0.088***	−0.007	−0.027**
	(0.0072)	(0.0099)	(0.0138)	(0.0129)	(0.0126)
wave 3	−0.001	0.007	0.031**	−0.010	−0.123***
	(0.0072)	(0.0087)	(0.0131)	(0.0125)	(0.0120)
wave 4	−0.013*	0.003	0.004	0.004	−0.059***
	(0.0076)	(0.0096)	(0.0139)	(0.0132)	(0.0131)
Constant	0.150***	0.142***	0.285***	0.393***	0.506***
	(0.0141)	(0.0144)	(0.0201)	(0.0245)	(0.0219)
Observations	9,536	9,532	9,484	9,164	9,537
R-squared	0.008	0.017	0.015	0.002	0.031
No. of Individuals	2,891	2,891	2,891	2,880	2,891

Note: Robust standard errors clustered at individual level in parentheses; *** p < 0.01, ** p < 0.05, * p < 0.1.

### Exposure definition and outcome measures

The primary exposure variable was the onset of a chronic disease diagnosis, defined as a self-reported physician diagnosis by survey respondents of cancer, diabetes, or cardiovascular disease between two survey waves. Cardiovascular disease included conditions related to the vascular, heart, and blood circulatory systems. A binary indicator variable, “health shock” was created for each person-wave, coded as 1 if a respondent reported being diagnosed with any of the three conditions in that wave, and 0 otherwise. We excluded individuals with previous diagnoses at baseline to isolate behavioral effects associated with incident diagnoses, rather than longer-term adjustments after years of illness. In robustness checks, we further disaggregated the health shock variable into separate indicators for cancer, diabetes, and cardiovascular disease to explore heterogeneous effects by disease type. Respondents with multiple concurrent diagnoses were included in each relevant disease-specific indicator rather than restricted to a single, mutually exclusive category, since doing so would reduce statistical power and obscure disease-specific effects; comorbidities were not entered as additional regressors in the main specification, as this would make the disease-specific estimates less distinguishable from one another. As an additional robustness check, we re-estimated the models with hypertension — the most common comorbid condition in the sample — included as a control variable, and found results similar in sign, significance, and effect size to the main specification.

We examined five primary outcome variables representing key modifiable health behaviors. These included: current smoking status (binary: 1 = currently smokes; 0 = otherwise), current alcohol consumption (binary: 1 = currently consumes alcoholic drinks; 0 = otherwise), physical activity (binary: 1 = exercises at least 150 minutes per week; 0 = does not meet this threshold). Physical activity was assessed against the 2020 WHO guidelines, which recommend at least 150–300 minutes of moderate-intensity or 75–150 minutes of vigorous-intensity aerobic activity per week. The measure was derived from respondents’ reported exercise frequency and average session duration. Normal body mass index (BMI) was calculated from self-reported weight and height and coded as a binary variable equal to 1 if BMI falls within the normal range of 18.5 upto 23 kg/m², a threshold com adjust for time-varying monly used in regional analyses; and waist obesity (binary: 1 = waist circumference exceeds 32 inches for women or 36 inches for men, based on winsorized waist circumference values (winsorized at the 1st and 99th percentiles); 0 = otherwise). All outcome variables were measured in each wave and treated as dependent variables in separate regression models.

### Covariates and potential confounding

To adjust for time-varying confounders, we included several covariates in our models. These included log-transformed personal income, a combined activities of daily living (ADL) score, and marital status. Personal income was log-transformed to reduce skewness. Household income or average household income per household member would better capture household economic resources, but these measures were available in only three of the four survey waves. Therefore, personal income was used as a proxy for economic resources. The ADL score was based on four of the six standard ADL domains collected by the survey—bathing/showering, brushing teeth, dressing, and eating—with lower scores indicating greater independence. These variables were selected based on their known associations with both chronic disease and health behaviors in prior literature.

The main empirical strategy employed is a fixed effects panel regression model inspired by generalized difference-in-difference framework, which estimates within-person changes in health behavior outcomes before and after the occurrence of a health shock. This approach controls for all unobserved, time-invariant characteristics that might confound the relationship between illness diagnosis and behavior change. Variables such as sex, years of education, or region are absorbed by individual fixed effects when they do not vary within individuals over time.

### Statistical analysis

The two-way fixed effects model specification was:


$$\begin{array}{c} Y_{it}=\beta_{\text{0}}+\beta_{\text{1}}{HealthShock}_{it}+{\gamma X}_{it}+\alpha_{i}+\lambda_{t}+\epsilon_{it} \end{array}$$

where *Y_it_* represents the health behavior of individual *i* at time *t*, *HealthShock*_*it*_ is the binary indicator for having diagnosed with the condition(s) at time t (including new and ongoing diagnosis), *X*_*it*_ is the vector of time-varying covariates, *α*_*i*_ captures individual fixed effects, and *λ*_*t*_ represents wave fixed effects to control for national trends and policy shifts over time. The coefficient of interest, *β*_1_, estimates the average change in health behavior following diagnosis, controlling for other factors. Robust standard errors were clustered at the individual level to account for within-person correlation.

To further explore heterogeneity by disease type, we estimated separate models for each condition—cancer, diabetes, and cardiovascular disease. This was based on prior literature suggesting that the salience of different conditions may influence behavior differently; for example, heart attacks may trigger more immediate lifestyle changes than asymptomatic diabetes. We also estimated interaction models for sex differences (Female × Shock) for alcohol outcomes, consistent with our theoretical discussion and known gender differences in drinking prevalence. Two additional binary alcohol outcomes were examined. Heavy drinking was defined as consuming more than 5–6 cans of beer or 5–6 glasses of wine or spirits per drinking occasion, based on the HART survey response categories. Frequent drinking was defined as reporting alcohol use at least 5–6 times per week.

The data used in this study were de-identified, with direct personal identifiers removed before access by the authors. Certain variables used in the analysis, including detailed health-status information, are classified by HART as sensitive data and were accessed in accordance with HART's controlled-access procedures and applicable data-use conditions. The original HART study received ethical approval from the Ethics Committee in Human Research, National Institute of Development Administration (ECNIDA), and respondents provided written informed consent at the time of the original data collection. This methodological approach leverages panel data to estimate within-person associations between chronic disease diagnosis and subsequent health behaviors, while addressing several limitations of cross-sectional and retrospective studies. The fixed effects framework allows us to isolate within-person time-invariant characteristics and contributes novel evidence from a developing country context, where the burden of chronic illness is rising but empirical research on post-diagnosis behavior remains limited. We followed the STROBE (Strengthening the Reporting of Observational Studies in Epidemiology) guidelines for transparent reporting of observational studies, ensuring complete disclosure of sample selection, model specifications, and robustness checks. All statistical analyses were conducted using Stata 18, with significance levels set at *p* < 0.05.

## Results

Table 1 presents descriptive statistics across four survey waves, comprising 9,577 person-wave observations. The number of respondents per wave ranged from 1,857–2,891. The proportion reporting at least one of the three chronic diseases was 0.0% in 2015, 9.0% in 2017, 14.2% in 2020, and 16.5% in 2022. Across pooled person-wave observations, the mean age was 68.43 years (SD 11.88), 53.2% were female, and 50.8% lived in rural areas. Marital-status proportions were 29.1% widowed, 5.5% single, 2.9% divorced, and 1.6% separated; approximately 60.9% were married. Secondary education and diploma-level education or higher accounted for 14.1% and 4.3%, respectively, while 5.7% had no education.

New participants were recruited into HART in the second wave. The analytic sample was therefore largest in wave 2, combining respondents retained from wave 1 and newly eligible respondents who also participated in at least wave 3. This partly explains why mean log personal income bacames lower in Wave 2.

Across pooled person-wave observations, 13.6% reported current smoking, 15.7% reported current alcohol use, and 25.2% met the physical activity threshold. The proportions with normal BMI and waist obesity were 39.5% and 37.7%, respectively (Table 1).

In the pooled models, diagnosis of any of the three chronic diseases was not significantly associated with current smoking, current drinking, meeting the exercise-duration threshold, normal BMI, or waist obesity (Table 2). Log personal income was positively associated with current smoking and drinking and negatively associated with waist obesity. Higher ADL scores were negatively associated with current smoking, current drinking, and meeting the exercise-duration threshold.

In disease-specific models, cancer and cardiovascular disease diagnoses were not significantly associated with any of the five outcomes at the 5% significance level (Table 3). Diabetes diagnosis was associated with a 3.6 percentage-point reduction in the probability of current drinking (p < 0.05), with no significant associations for the other outcomes.

**Table 3:** Associations between disease-specific diagnosis indicators and health behaviors (three separated sets of regressions).

			Enough	Normal	Waist
VARIABLES	Smoking	Drinking	Exercise	BMI	Circumference
cancers	−0.037	−0.058	−0.026	0.057	−0.047
	(0.0336)	(0.0391)	(0.0528)	(0.0544)	(0.0511)
diabetes	−0.009	−0.036**	0.031	0.004	−0.027
	(0.0149)	(0.0183)	(0.0278)	(0.0275)	(0.0276)
cardiovascular	−0.000	−0.007	−0.003	0.053*	−0.013
	(0.0226)	(0.0229)	(0.0315)	(0.0314)	(0.0309)
Observations	9,536	9,532	9,484	9,164	9,537
No. of Individuals	2,891	2,891	2,891	2,880	2,891

Note: Covariate coefficients included as in Table 2 are omitted here. Robust standard errors clustered at the individual level are in parentheses; *** p < 0.01, ** p < 0.05, * p < 0.10.

Diabetes diagnosis was associated with reductions of 3.6 percentage points in current drinking (p < 0.05) and 3.5 percentage points in frequent drinking (p < 0.01), but was not significantly associated with heavy drinking (Table 4). Cancer diagnosis was associated with an 8.0 percentage-point reduction in frequent drinking (p < 0.01), with no significant associations for current or heavy drinking. Cardiovascular disease diagnosis was not significantly associated with any of the three drinking outcomes.

**Table 4:** Associations between disease-specific diagnoses and drinking behaviors (three separated sets of regressions).

VARIABLES	Drinking	Heavy Drinking	Frequent Drinking
cancers	−0.058	−0.037	−0.080***
	(0.0391)	(0.0314)	(0.0285)
diabetes	−0.036**	−0.011	−0.035***
	(0.0183)	(0.0117)	(0.0123)
cardiovascular	−0.007	−0.001	−0.013
	(0.0229)	(0.0149)	(0.0183)
Observations	9,532	9,532	9,532
No. of Individuals	2,891	2,891	2,891

Note: Covariate coefficients included as in Table 2 are omitted here. Robust standard errors clustered at the individual level are in parentheses; *** p < 0.01, ** p < 0.05, * p < 0.10.

In the sex-interaction models, diabetes diagnosis was associated with reductions in current drinking, heavy drinking, and frequent drinking among men (all p < 0.05; Table 5). The female-by-diabetes interactions were statistically significant for heavy drinking at the 5% level but not for current or frequent drinking. However, the Wald test based on linear combination of these coefficients indicate no statistically significant associations among women (all p > 0.10). Cardiovascular disease diagnosis was not significantly associated with drinking outcomes in either sex.

**Table 5:** Sex differences in associations between disease-specific diagnoses and drinking behaviors.

VARIABLES	Drinking	Heavy Drinking	Frequent Drinking
CANCER			
cancers	−0.151**	−0.148**	−0.139**
	(0.0590)	(0.0609)	(0.0550)
female#cancers	0.153**	0.180***	0.096*
	(0.0712)	(0.0623)	(0.0569)
DIABETES			
diabetes	−0.064**	−0.051**	−0.059**
	(0.0324)	(0.0226)	(0.0231)
female#diabetes	0.053	0.075***	0.045*
	(0.0355)	(0.0233)	(0.0242)
CARDIOVASCULAR			
cardiovascular	−0.023	−0.008	−0.023
	(0.0377)	(0.0255)	(0.0320)
female#cardiovascular	0.033	0.016	0.022
	(0.0430)	(0.0276)	(0.0336)
Observations	9,532	9,532	9,532
No. of Individuals	2,891	2,891	2,891

Note:Covariate coefficients included as in Table 2 are omitted here. Robust standard errors clustered at the individual level are in parentheses; *** p < 0.01, ** p < 0.05, * p < 0.1

As for cancer diagnosis, the health shock was associated with reductions in all three drinking outcomes among men (all p < 0.05; Table 5). Although the female-by-cancer interactions were statistically significant at the 5% level for all three outcomes, no significant changes were observed among women (all Wald tests p > 0.1; available upon request).

## Discussion

This study adds longitudinal evidence from Thailand to the limited literature on behavioral responses to chronic disease diagnosis in middle-income Asian settings. Using four waves of the HART survey, we found that the onset of diabetes or cancer among Thai adults was associated with modest and selective reductions in alcohol consumption, while no significant within-person changes were observed in smoking, physical activity, or obesity-related indicators. These findings underscored the limited behavioral responsiveness to diagnosis alone and highlighted the importance of structural, psychosocial, and cultural factors in sustaining behavior change after chronic disease onset.

These results reinforced calls for integrating multi-level frameworks that link individual cognition with social and institutional determinants of behavior. Models such as the Socio-Ecological Model [25] and Strong Structuration Theory [26] emphasize that individual decisions are embedded within relational and environmental contexts that shape both capability and opportunity for change. In the Thai context, health behaviors unfold at the intersection of personal efficacy, community norms, and health system engagement, illustrating what Glass and McAtee [27] describe as “risk regulation through structure.” Our findings are consistent with the view that effective secondary prevention may depend depended not only on individual motivation but also on the alignment of behavioral interventions with broader social and institutional infrastructures.

### Selective change and the limits of the “teachable moment”

Our findings mirrored global evidence that chronic disease diagnosis can act as a partial teachable moment—a psychological event that increases awareness [28]. Studies across North America, Europe, and Asia consistently report selective and short-lived improvements following diagnosis [12,29,30]. In line with previous research, our study found reductions in alcohol consumption associated with diabetes diagnosis, but no statistically significant associations for the other primary outcomes.

Behavior-specific responsiveness has been documented across multiple settings. For example, in the English Longitudinal Study of Aging, people with type 2 diabetes reduced smoking but showed no diagnosis-specific improvements in diet or physical activity [29]. Similarly, Canadian longitudinal data showed reductions in smoking in most diagnostic groups; people with diabetes also reduced excessive drinking and increased physical activity, with patterns varying across conditions [30]. In Asia, Korean evidence showed that even after type 2 diabetes onset, younger or lower-income men are likely to continue smoking [3]. Our results suggested similar behavioral inertia in Thailand, reflecting how socioeconomic and environmental constraints moderate the “awareness-to-action” pathway.

### Alcohol, and not exercise or weight, responding to diagnosis

The decline in alcohol use—especially among men with diabetes—may reflect clinical advice or differences in the feasibility of changing particular behaviors, although these explanations were not tested. Clinical recommendations do not necessarily translate into lifestyle change, as observed among US adults with diabetes [31]. The capability, opportunity, and motivation framework emphasizes that behavior change depends on more than motivation alone [32]. A cross-sectional analysis of the Thai Cohort Study associated heavy drinking with higher odds of selected NCDs and reported lower drinking prevalence among women [33].

By contrast, exercise and weight-related indicators were not significantly associated with diagnosis at the 5% level. The Health Belief Model identifies perceived barriers and benefits as relevant to behavior change [34]. Qualitative research among adults with type 2 diabetes in Bangkok also describes how people reconcile illness with a sense of normality [35]. Our findings suggest that diagnosis alone may be insufficient to produce broad behavioral improvements and highlight the potential importance of sustained health-system engagement.

### Gendered and socioeconomic patterns in post-diagnosis behavior

The sex-related patterns observed in our analysis suggested reductions in several drinking outcomes among men, while the evidence of corresponding reductions among women was less conclusive. Men are far more likely to drink or smoke, while women’s engagement in these behaviors is culturally constrained [33]. Consequently, women had less “room to change.” This asymmetry also underscored how social norms shaped perceived behavioral control—a key mechanism in the Theory of Planned Behavior [36].

Educational or income-related disparities in behavior change were less pronounced in our study, possibly due to cohort effects. In the multicohort study across 17 countries, socioeconomic gradients in post-diagnosis behavior were striking: low-income individuals were far more likely to continue smoking and remain inactive after diagnosis than high-income peers [2]. However, some subgroups within that study, such as China, did not show significant socioeconomic gradients in health behaviors.

Our results aligned with qualitative and ethnographic accounts emphasizing cultural rationalization of chronic disease in Thailand. Naemiratch and Manderson [35] describe how diabetes patients conceptualize their illness. This framing may limit the perceived need for drastic behavior change and encourage selective adjustments (e.g., reduced alcohol) that are socially acceptable and symbolically sufficient [35]. Moreover, cross-sectional Thai data reveal that even among older adults with hypertension and diabetes, awareness of chronic disease complications can coexist with moderate understanding of the benefits and barriers to behavioral change [37].

In the broader regional literature, a review reported poor glycemic control in more than half of research participants in the studies it discussed and identified multiple associated factors [38]. Likewise, longitudinal evidence from Thailand indicates that having three or more chronic conditions is associated with higher odds of incident and persistent depressive symptoms among middle-aged and older adults [39].

### Diagnosis without support: structural and systemic constraints

Comparative research on diabetes and stroke services in Malaysia, Sri Lanka, and Thailand highlights the need to reorient health services toward ongoing chronic care [40]. Our findings suggested that diagnosis was not consistently associated with improvements across multiple health behaviors, underscoring the potential value of structured post-diagnosis support. This is consistent with the self-management literature, which emphasizes that feedback, social support, and problem-solving training are key determinants of sustained adherence [31,41]. Thai research has also identified factors associated with health literacy and self-care behaviors among patients with type 2 diabetes [42].

Our findings supported the argument that diagnosis should serve as the starting point for structured behavior-change pathways, not the endpoint of clinical engagement. Evidence from Thailand and International reviews suggest potential benefits of mobile health interventions for selected chronic conditions [43–45]. Evidence from a Thai quasi-experimental study suggests potential benefits of integrated health-promotion programs and supportive peers for improving selected health behaviors among older adults. Further research is examining peer health-coaching approaches for chronic disease prevention [46,47]. Interventions should therefore also address social patterning of change, targeting low-income and rural populations where environmental constraints limit sustained improvement—a conclusion consistent with prior evidence on social inequalities in behavioral adaptation [48].The effectiveness of these approaches in supporting sustained post-diagnosis change in Thailand requires further evaluation.

A major strength of this study was its within-person longitudinal design, which isolated behavioral changes temporally linked to diagnosis, controlling for unobserved individual characteristics. This design allowed the study to estimate within-person associations between diagnosis and behavioral outcomes after accounting for time-invariant individual characteristics and observed time-varying covariates. However, the study was not without limitations. Disease diagnoses and behavioral outcomes were self-reported, introducing potential measurement error. The two- to three-year survey intervals constrained the ability to observe short-term responses or relapse. We assumed that reported health behaviors reflected respondents’ status at the time of survey; consequently, any observed behavioral changes were interpreted as occurring after the realisation of the health shock, though the exact timing within each survey interval could not be determined. Four waves of data limited the ability to examine prediagnosis behavioral trends, and sample attrition may introduce selection bias, and sample attrition may introduce selection bias. Antidiabetic medications may also influence alcohol consumption. Because HART did not provide information on the types of medications taken, we could not distinguish associations with diagnosis from potential medication effects.

Hypertension was not included in the main health-shock definition. Although hypertension is clinically important and is often managed through lifestyle modification, prior studies suggest that hypertension diagnosis does not always produce consistent behavioral responses across domains such as physical activity, smoking,alcohol use, and diet [49,50]. Conceptually, hypertension is commonly asymptomatic, gradual in onset, and may not be perceived by respondents as an acute or salient health shock in the same way as cancer, diabetes, or cardiovascular disease. This distinction is important because previous studies have treated hypertension as a health shock most convincingly when using biomarker-based blood pressure thresholds, such as systolic or diastolic blood pressure cut-offs, to identify first-ever diagnosis or health-information shocks [49,51,52]. In addition, the HART data rely on self-reported physician diagnosis rather than biomarker-based blood pressure thresholds or exact diagnostic dates. This limits our ability to distinguish newly detected hypertension from previously existing but undiagnosed or inconsistently reported hypertension. We therefore defined health shocks using incident reports of cancer, diabetes, and cardiovascular disease, which were more directly aligned with the study’s focus on salient chronic disease onset and selective behavioral response. Nevertheless, we acknowledge that some previous studies have included hypertension within broader health-shock definitions, particularly in analyses of preventive behavior change among middle-aged and older adults [6].

Future research should therefore incorporate objective biomarkers, refined event timing, longer longitudinal panels, and attrition-robust analytic techniques to strengthen causal inference. Nevertheless, these findings underscored a broader theoretical and policy insight: diagnosis represented an initiating signal rather than a sufficient condition for behavioral transformation. Sustainable health behavior change in aging societies will depend not on awareness alone but on the institutional, cultural, and social infrastructures that enable individuals to act upon that awareness.

## Conclusions

Among middle-aged and older adults in Thailand, chronic disease diagnosis was associated with selective changes in current alcohol use, particularly among men, rather than broad improvements across health behaviors. These findings suggest that diagnosis alone may be insufficient to prompt comprehensive behavioral change. Alcohol screening, brief intervention, and follow-up within diabetes and oncology care warrant consideration, particularly for older men. Broader behavioral risks may require sustained clinical and community support tailored to patients’ circumstances. Because the analysis was observational and the timing of diagnosis and behavior change within survey intervals was uncertain, these associations should not be interpreted as definitive causal effects. Future research should evaluate which forms of post-diagnosis support produce sustained improvements.
